# Online Signature Biometrics for Mobile Devices

**DOI:** 10.3390/s24113524

**Published:** 2024-05-30

**Authors:** Katarzyna Roszczewska, Ewa Niewiadomska-Szynkiewicz

**Affiliations:** Institute of Control and Computation Engineering, Warsaw University of Technology, Nowowiejska 15/19, 00-665 Warsaw, Poland; katarzyna.roszczewska.dokt@pw.edu.pl

**Keywords:** biometrics, signature recognition, mobile devices, convolutional neural networks, artificial intelligence

## Abstract

This paper addresses issues concerning biometric authentication based on handwritten signatures. Our research aimed to check whether a handwritten signature acquired with a mobile device can effectively verify a user’s identity. We present a novel online signature verification method using coordinates of points and pressure values at each point collected with a mobile device. Convolutional neural networks are used for signature verification. In this paper, three neural network models are investigated, i.e., two self-made light SigNet and SigNetExt models and the VGG-16 model commonly used in image processing. The convolutional neural networks aim to determine whether the acquired signature sample matches the class declared by the signer. Thus, the scenario of closed set verification is performed. The effectiveness of our method was tested on signatures acquired with mobile phones. We used the subset of the multimodal database, *MobiBits*, that was captured using a custom-made application and consists of samples acquired from 53 people of diverse ages. The experimental results on accurate data demonstrate that developed architectures of deep neural networks can be successfully used for online handwritten signature verification. We achieved an equal error rate (EER) of 0.63% for random forgeries and 6.66% for skilled forgeries.

## 1. Introduction

A handwritten signature is probably the oldest and the most socially accepted method of biometric authentication of an individual [1]. With the development of technology, signature acquisition techniques were improved, and a new branch was developed—online handwritten signatures. An image of the signature was replaced with a sequence of x-y coordinates in time, along with various associated attributes like pressure, stylus pivot, and acceleration. Despite that, the handwritten signature’s most severe imperfection stays the same—users’ tidiness of handwriting causes a huge fluctuation between each sample. Due to this fact, samples of the same person can vary in duration, position, or shape, making identification and verification problems so complex. All these problems are widely discussed in [2] providing an overview of biometrics methods. Luckily, the current level of technological development made it possible to create new data processing methods that improved devices’ computing power and enabled the implementation of artificial neural networks on devices such as cloud servers, personal computers, or even mobile phones [3]. However, the scientific literature survey shows that there is still relatively little work on verification methods that can be used on a mobile phone. Most techniques require the use of specialized tablets.

In our research, we have focused on online handwritten signature verification on mobile phones. Attention has been paid to methods based on machine learning techniques. To the best of our knowledge, this article is the first to describe the use of convolutional neural networks in signature recognition utilizing the dataset collected with mobile devices with pressure values in each point. Our research concluded that exploiting mobile phones with pressure sensors is quite a novelty in biometry. Due to the number of mobile device models with so-called “Force Touch” still increasing, questions about the accuracy of their sensors appeared. The results of the complete studies on the precision of pressure sensors are described in the report [4]. The authors conducted the experiments with pressure sensors of four mobile phones: Apple iPhone 6s, iPhone 7, Huawei Mate S Premium, and ZTE AXON Mini. The results confirmed that the accuracy of measurements of tested sensors is good enough for acquiring signature data enhanced with pressure characteristics. The report convinced us that including pressure characteristics in handwritten online signatures entered on a mobile device could affect their verification results.

To sum up, the main contributions of this paper are:

- A method for preprocessing online signatures gathered on mobile phones into the valuable form for verification systems;
- Two custom-made classifiers (SigNet and SigNetExt) using convolutional neural networks for signature recognition on mobile phones;
- A comprehensive study comparing online signature recognition performance based on the commonly used pre-trained VGG-16 model for image recognition and our SigNet and SigNetExt models.

The main innovation is a novel light convolutional neural network architecture that uses only coordinates of points and pressure values at each point collected with a mobile device for signature verification. Our solution does not require additional measurements that can be collected with specialized tablets for signature recognition.

The rest of the paper is structured as follows. Section 2 presents the survey of the online handwritten signature verification methods, the usage of mobile devices for signature acquisition, and mobile cross-device interoperability in signature recognition. Artificial neural network architectures for signature verification are overviewed in Section 3. Section 4 describes the training datasets preprocessing, evaluation metrics, and results of training and validation of proposed neural network models. The performance evaluation of three network models is presented and discussed in Section 5. Finally, Section 6 concludes the paper and highlights future research directions.

## 2. Related Work

The problem of online handwritten signature recognition and verification is not trivial. However, various methods have been developed and described in the literature. Generally, they can be can be broadly classified into three classes:

- Nonparametric methods, e.g., Dynamic Time Warping (DTW);
- Parametric methods, e.g., Hidden Markov Models (HMM);
- Parametric methods with unknown parameter numbers, e.g., artificial neural networks.

DTW measures the similarity between two temporal sequences, which may vary in speed. Moreover, a so-called “warping path” is determined. Warping according to this path may align the two sequences in time. Thus, DTW is commonly used when two signatures are matched in handwritten signature verification. In the original DTW, the warping path is determined based on all signature features. Putz-Leszczynska in [5] describes the slightly modified DTW, in which the warping path is calculated only based on positional coordinates. Thus, the difference ratio between the two signatures was calculated from coordinates x and y and the values of pressure and pivot of the stylus. The method was tested on handwritten signatures collected by the author. The results of signature verification were as follows: 1.04% False Acceptance Rate (FAR), 1.86% False Rejection Rate (FRR). The lower the FAR and FRR, the more effective the biometric method.

DTW was also used to compute similarity metrics of signatures in [6]. Sadak et al. proposed an approach to verifying signatures, in which they analyzed the friction sound that arises from the contact between paper and pen. The sound was recorded using two different mobile phone models. The strength envelopes of sound signals were compared. The reported results were as follows: Equal Error Rate (ERR) values from 8.14% to 16.61% for calculations with signer-specific thresholds, EER values from 15.29% to 28.24% for calculations with a single threshold for all users. The experiments were conducted with users of different ages using various types of paper, pens, and mobile phones.

The authors of [7] used Hidden Markov Models (HMMs) to model a handwritten signature. They tested their method on the MCYT database [8], which contains signatures acquired from 330 people. The research focused on the influence of sampling frequency and interpolation methods on online handwritten signature recognition accuracy. The comparative study for several frequencies and two interpolation methods, i.e., line interpolation and Catmull-Rom, is described and discussed in [7]. The authors claim they obtained the results with a 7.35% EER for skilled forgeries at best. EER is a value of error where the false rejection and acceptance rates are equal. The lower this value, the more effective the biometric method.

Artificial neural networks have received much attention recently. Many researchers investigate the feasibility of using these techniques for handwritten signature recognition. Two main approaches can be distinguished, i.e., the application of perceptron networks and Convolutional Neural Networks (CNNs). Online signature recognition utilizing perceptron neural networks is discussed in paper [9]. The authors focused only on the characteristics of coordinates in the domain of time. Preprocessing consisted of duration scaling to 512 points using double linear interpolation, Fourier transforms (to reduce shifts caused by different starting points), and Mellin transform, independent of the scaling process. The accuracy of signature recognition was tested on data collected in the SVC2004 database [10]. The performance of two classifiers, i.e., the perceptron neural network and linear classifier, was compared with Principal Component Analysis (PCA). The best values of the EER metrics were 6.70% for the PCA-based classifier and 3.00% for the neural network model, respectively. Another neural network technique for online handwritten signature verification is described and evaluated in [11]. The author developed and compared the effectiveness of four binary classifiers for verifying the identity of two signatures of the same person. They employed for signatures classification:

- x and y coordinate differences;
- Linear classifier;
- Multilayer perceptron;
- Convolutional neural networks.

The Anoto Pen stylus [12] and a special sheet of paper were used to gather and create datasets. It has the same characteristics as the ones gathered from a regular graphic tablet. The authors used a custom database of 46 classes, each with seven signature samples for training and testing purposes. All signatures were preprocessed with duration scaling and linear interpolation. The dataset was divided into training, validation, and testing subsets (five training images, one validation image, and one test image for each class). Unfortunately, this paper does not provide information about skilled forgeries. All tests were performed for random forgery verification only. The best results presented were achieved for the classifier based on CNNs with an EER of around 3.00%.

In [13], the authors investigated the feasibility of using Recurrent Neural Networks (RNNs) for handwritten signature verification. They describe the method employing the Time-Aligned Recurrent Neural Networks (TA-RNNs), a significant advancement in the field of signature verification. TA-RNNs leverage the synergistic between DTW and recurrent neural networks to enhance the robustness and accuracy of signature authentication systems. TA-RNNs demonstrate superior performance in distinguishing genuine signatures from fraudulent attempts by effectively aligning time sequences and extracting meaningful features. Moreover, the authors point out in [13] the challenges of handwritten signature verification, the difficulties posed by the scarcity of publicly available datasets, and the challenges in assessing the efficacy of emerging techniques. To reduce these limitations, they designed and developed an innovative online repository meticulously curated to collect diverse handwritten signatures (DeepSignDB). This repository offers a wealth of data for exploration and experimentation in signature verification and is commonly used by many researchers. The authors provide a comprehensive exposition of the DeepSignDB database, elucidating key aspects such as acquisition modalities, impostor typologies, and dataset specifications. This detailed overview facilitates a nuanced understanding of the dataset and empowers researchers to conduct thorough analyses and draw meaningful insights from their investigations. Finally, Tolosana et al. present a rigorous and standardized experimental framework to facilitate equitable comparisons among various methodologies. This framework aims to mitigate the confounding factors often encountered in comparative studies, enabling researchers to ascertain their approaches’ true efficacy and generalizability.

A comprehensive overview and comparative study among handwritten signature verification methods using machine learning is presented in [3]. Hashim et al. compare and discuss the research results reported in more than twenty research papers from 2012 to 2022. They list available online and offline datasets and describe feature extraction and verification methods. Moreover, they highlight the advantages and limitations of machine learning application techniques in signature verification systems.

To sum up, most research papers on handwritten signature biometrics focus on various methods and techniques for signature verification on specialized tablets for signature recognition. The literature offers relatively few reports of signature biometrics on a mobile device, while every mobile phone and tablet contains the abilities of a signature-capturing device. The following papers focus on signature verification on mobile devices. Sae-Bae and Memon in [14] describe a system for online user authentication based on a handwritten signature on a mobile device. The authors use statistical representations of online signatures. A histogram represents empirical distributions of signature features. Signature preprocessing consists of two steps, i.e., time normalization and signature stroke concentration. Next, histogram features are extracted from the signatures of each signer, and a unique signature pattern is created for verification. The verification process consists of the following steps: (i) a new sample preprocessing, (ii) histogram features extracting, and (iii) calculating similarity to the template. A series of experiments were conducted with MCYT [8] and SUSIG [15] datasets to demonstrate the effectiveness of this method. Test results for the MCYT dataset depended on the number of user samples used and were as follows:

- Skilled forgeries: EER = 4.02% (five samples), EER = 2.72% (ten samples);
- Random forgeries: EER = 1.15% (five samples), EER = 0.44% (ten samples).

Test results for the SUSIG dataset and five samples were as follows:

- Skilled forgeries: EER = 6.08% (five samples);
- Random forgeries: EER = 2.94% (five samples).

Next, a series of experiments were conducted on the dataset created by the authors. They have developed an experimental platform in HTML5 for collecting data concerning handwritten signatures. The data collection was divided into six sessions, with five samples per participant drawn in uncontrolled and unsupervised conditions. The results of a cross-session verification outperformed the previous ones, EER = 2.89%.

Fakhiroh et al. describe in [16] another system for handwritten signature verification on mobile devices. They used offline signatures and convolutional neural networks to develop a successful algorithm for signature recognition. The reported verification accuracy was 84.45% and was increased to 88.89% after the CNN network adjusted to the problem. The authors integrated the CNN model into a mobile application designed for real-time signature verification. The critical functions of this application include capturing signature images using the device’s camera, selecting images from the device’s gallery, or utilizing cropped document images for verification purposes. The application’s user-friendly interface facilitates ease of use, guiding users through signature verification. In their research, the authors note the challenges of limited training data availability and the potential loss of feature information during training. To address these challenges, they suggest applying data augmentation techniques to increase the training dataset and, finally, to improve the robustness and accuracy of trained models.

Training data sets contain signatures collected on various devices, often other than the device on which the signature is verified. Hence, the data must be preprocessed. The authors of the paper [17] performed a complete study on cross-device interoperability and performance evaluation of the quality of signature verification on various devices. Data acquired for research purposes was captured with eight devices, i.e., six mobile ones (tablets and PDAs) and two Wacom digitizers [18]. The experimental data were collected from 43 volunteers aged above 16. The data collection was divided into three sessions, each with 60 signatures. Values of X and Y coordinates and a time stamp were gathered for each sample. The collected data was preprocessed using the following techniques, i.e., linear interpolation, signal filtering, and normalization. The authors used the DTW method for signature verification.

Next, two experiments were conducted:

- An intra-device performance study;
- An intra-/inter-modality analysis where modalities depend on the data acquisition technique, i.e., stylus or finger-based.

In inter- and intra-modality experiments, samples from a given device (using finger or stylus-based) were used with the biometric reference from the other finger or stylus-based device, respectively. The cross-device study shows a few correlations between EER and some device parameters such as screen size, area of signing space, screen resolution, or visual feedback to the user. The best efficiency (EER = 0.19%) was achieved for iPad2–a finger-based device with the most giant screen in the group. The best result for stylus-based devices (EER = 0.58%) was obtained for the Samsung Galaxy Note. The authors hypothesize that the visual feedback and screen resolution most significantly influence the accuracy of signature recognition. Finally, the authors claim that the signing performance using stylus-based and finger-based devices is generally similar on average. However, finger-based signing was considered less comfortable for users.

In conclusion, while the problem of identity recognition using handwritten signature analysis is well-described, there needs to be a comprehensive research study on deep learning in online signature verification on mobile devices. To fill this gap, we developed and compared two various neural network models for signature classification based on real data.

To sum up, handwritten signature verification is one of the behavioral biometrics that can be used to improve the security of mobile devices and personal computers. Other approaches to authentication include the use of sensors mounted on the device. Smartphone motion analysis supporting PIN-based authentication is proposed in [19]. Behavioral biometrics modalities such as keystroke and mouse dynamics often strengthen user authentication security in computer systems [20,21].

## 3. Convolutional Neural Networks for Handwritten Signature Verification

### 3.1. Convolutional Neural Network (CNN)

Convolutional neural networks allow the automatic detection of relevant information without human supervision [22]. They use convolution and pooling operations. In CNN, the input data is a multidimensional array, while in other simple artificial neural networks, it is one-dimensional. To prevent the padding problem, extending output is filled with zeros when the output differs in size from the input. CNN comprises a few convolutional layers with the Rectified Linear Unit activation function (ReLU). Each layer is a collection of filters with parameters w×h×d, where *w* denotes width, *h* height, and *d* depth, respectively. The number of activation maps at the output equals the number of filters in a single layer. The output from the convolutional layers is downsampled by using pooling layers. The pooling layer maintains the most relevant information while decreasing the input’s spatial size. In the aggregation process, a window of predefined size is shrunk to a single element (usually with an average or maximum value in a window). The part of the network responsible for input data classification follows convolutional layers. It comprises a few fully connected layers with the softmax function.

Convolutional neural networks are commonly used to classify diverse data from various domains, especially image recognition. The CNN architecture has already been used with good scores for handwritten signature recognition and verification, as is presented in Section 2. Therefore, we have developed and evaluated a novel method employing CNN for signature biometrics on a mobile phone.

The problem being solved is the authenticity of customer signature verification from an established set of *M* users. Our method aims to classify each input signature as the signature of the *m*-th user (m=1,…,M) or to determine that it is the signature of a person from outside the given set of users. Thus, the CNN model output for a given input signature is a vector of probabilities for m=1,…,M classes membership. Then, similarity values to signatures from classes m=1,…,M are compared to the acceptance threshold predefined in the method. The comparison result determines whether or not the acquired sample is of class *m*. So, it is probably the signature of the *m*-th user. We proposed and evaluated three multi-class classifiers to solve this problem. At first, we incorporated and adjusted the CNN model utilizing the VGG-16 architecture described in [23]. VGG-16 is widely used in image recognition. Next, we designed and tested a novel custom-made CNN architecture called SigNet and trained two models implementing this architecture.

### 3.2. VGG-16 Neural Network Architecture

At first, we incorporated and adjusted the CNN model utilizing the VGG-16 architecture described in [23]. The aim was to estimate probabilities of belonging to each input signature (i.e., each user) to m=1,…,M classes.

The VGG-16 network architecture was designed by the Visual Geometry Group at the University of Oxford and is described in [23]. The VGG-16 model trained on natural images is widely used in image recognition. In our approach, signatures are processed to images, so it was natural to check how models dedicated to images handle their recognition. Results of numerous studies presented in literature [24,25] confirmed that the VGG-16 model can be successfully used to solve classification tasks in various domains after fine-tuning and appropriate adaptation.

The VGG-16 network comprises 13 convolutional layers with the ReLU function. A max-pooling layer follows each of the two convolutional layers. Three fully connected layers with ReLU follow the convolutional section. The parameters of the layers are as follows:

- 64 filter kernels with a size of 3 × 3;
- 128 filter kernels with a size of 3 × 3;
- 256 filter kernels with a size of 3 × 3;
- 512 filter kernels with a size of 3 × 3.

The size of the input array is 224×224. The Stochastic Gradient Descent (SGD) optimizer [26] with momentum value m=0.9 was used for training purposes. The initial learning rate was equal to 0.0001. To prevent model overfitting, we added a dropout layer to the original network with a dropout value equal to 50%.

### 3.3. SigNet Neural Network Architecture

VGG-16 is a complex network, and its learning is time-consuming. Moreover, the input of our method is a set of images that feature little content. Most of each image is a background. In this case, it was natural to look for a simpler classifier that is not less effective than VGG-16 and requires smaller learning datasets and training time. Therefore, we have developed a novel custom-made light network model (SigNet) for verifying the authenticity of customer signatures from an established set of *M* users.

**Figure 1:**
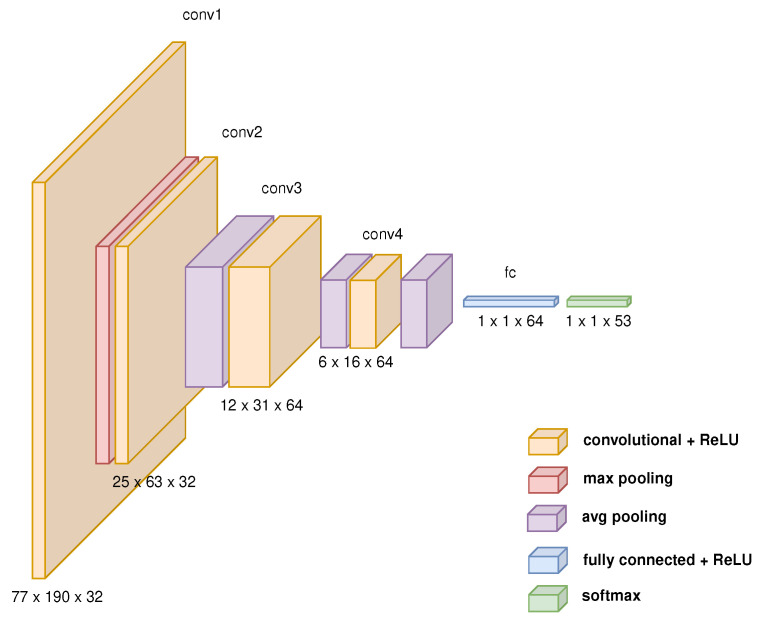
SigNet network architecture diagram.

The SigNet network architecture (Figure 1) comprises four convolutional layers with the ReLU function, pooling and softmax layers, and fully connected layers arranged in the following order:

- 1st convolutional layer with ReLU;
- Max pooling layer;
- 2nd, 3rd and 4th convolutional layer with ReLU, each followed by average pooling layer;
- One fully connected layer with ReLU;
- Softmax layer.

The parameters of the layers are as follows:

- 32 filter kernels with a size of 9 × 9;
- 32 filter kernels with a size of 7 × 7;
- 64 filter kernels with a size of 5 × 5;
- 64 filter kernels with a size of 6 × 16.

The size of the input array is 77×190.

We implemented our convolutional neural network using Matlab. We used the SGD optimizer with momentum value m=0.9. The initial learning rate equal to 0.05 was finally decreased to 0.0005.

Table 1 summarizes the parameters of both CNN architectures, i.e., SigNet and VGG-16.

## 4. Training and Validation

### 4.1. Database of Online Signatures

**Figure 2:**
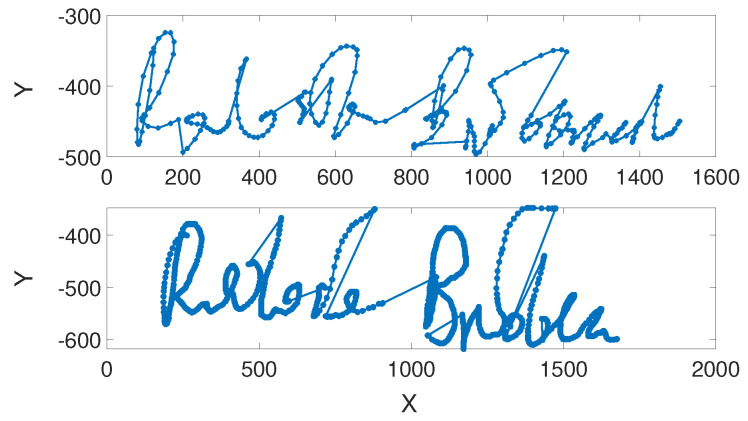
An example of a genuine signature (**top**) and its skilled forgery (**bottom**).

We trained and validated both classifiers on data from the multi-modal database, *MobiBits* [27], collecting samples from a group of volunteers. All signatures were acquired on the Huawei Mate S device using the Adonit Dash 2 stylus. This mobile phone is equipped with pressure sensors built inside the touchscreen. Due to this fact, points of each signature were evaluated with x and y coordinates (Figure 2) and the pressure value put on the touchscreen. A custom-made application was developed and used for data acquisition. The system asked users to enter a series of their original signature samples and saved the data locally on the device. Users could reset a sample attempt or start the session from the beginning.

Moreover, the application was used to capture skilled forgeries. Each user was asked to select a victim’s identification number. Then, the application displayed the genuine signature chosen for forging. The user had time to train a fake signature before entering the system.

**Figure 3:**
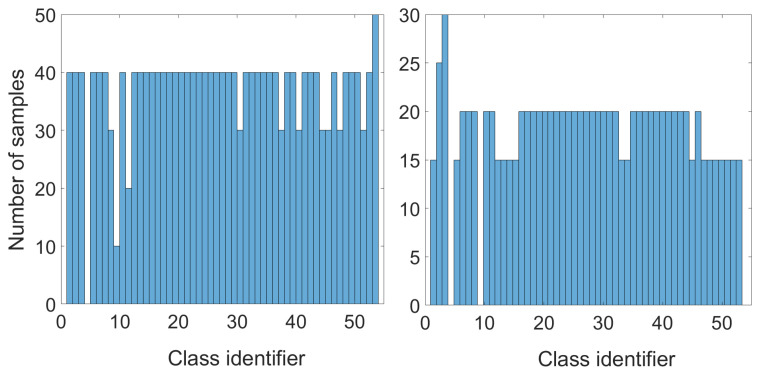
Number of samples for each class: genuine signatures (**left**) and skilled forgeries (**right**).

The dataset (DataSet) used in our research consisted of signatures acquired from 53 volunteers of diverse ages. Data collection was divided into three sessions (20 genuine signatures in the first session and 10 genuine signatures in the second and third). Moreover, each user was asked to enter 20 skilled forgeries. Finally, all collected samples were merged into a dataset with 53 classes. The distribution of genuine signatures and skilled forgeries for each class is presented in Figure 3. It should be noted that we did not include signature changes over time in our research.

Then, the DataSet was randomly divided into three subsets: TrainSet–training set (50% of DataSet), ValSet–validation set (25% of DataSet), TestSet–testing set (25% of DataSet).

### 4.2. Handwritten Signatures Preprocessing

**Figure 4:**
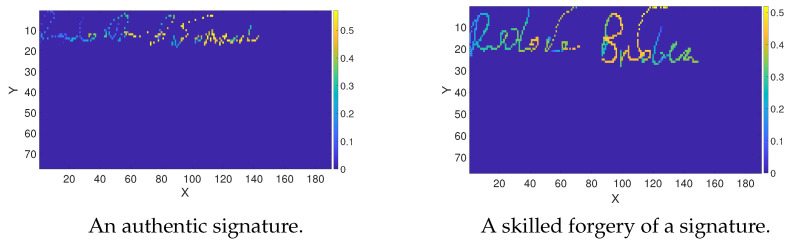
Handwritten signatures represented as images (pixels correspond to pressure values), scaled to the SigNet input data size.

Convolutional neural network models are incredibly efficient in image recognition. They successfully learn values in specific points of the image and their arrangement in space. Thus, the input data are images. Signature data samples collected with mobile devices are vectors of two coordinates *x* and *y*, and pressure values *p*. Therefore, to use the potential of CNN, we converted handwritten signatures into images with pressure values corresponding to the pixels. Figure 4 depicts the example signature representation after conversion.

Each image of a signature was resized using linear interpolation to fit both network architectures’ predefined input data sizes. In our research, the input data dimensions were 224×224 pixels for the VGG-16 network and 77×190 pixels for the SigNet network. In the VGG-16 model, the authors strictly defined the input size, which cannot be modified. In the case of SigNet, the input size was fitted to the sizes of samples acquired from volunteers and collected in the *MobiBits* database. A signature with maximum dimension determined the size of the SigNet input.

### 4.3. Evaluation Metrics and Results

Three network models were trained:

- SigNet: the SigNet network architecture trained on the dataset of 968 samples acquired using a custom-made application (the base training dataset).
- SigNetExt: the SigNet network architecture trained on the augmented dataset of 9680 samples. The base dataset was augmented by multiplying the base training dataset. Each sample was transformed with 5 random shifts and 20 rotations (from $-10^{\circ }$ to $9^{\circ }$) for each shifted image.
- VGG-16: the VGG-16 network architecture trained on the augmented dataset of 968 samples. Each sample was transformed with shifts randomly selected from the range [−10px,10px] in *x* and *y* coordinates and pivots randomly selected from $\left[-15^{\circ },15^{\circ }\right]$ for each shifted image. The network was trained with slightly different images in each epoch. Thus, the size of the training dataset was not increased, but it was randomly modified.

To reduce variability and generalize the results, we performed multiple rounds of cross-validation using randomly selected subsets, i.e., TrainSet, ValSet, and TestSet of data from DataSet. Our research averaged the validation and testing results over 20 rounds to estimate a given classifier’s predictive performance. Hence, 20 triples from TrainSet, ValSet, and TestSet were randomly selected for the training, validation, and testing phases.

Commonly used criteria were taken to assess the quality of classifiers in validation and testing procedures.

- EER—a statistic commonly used in performance evaluation of verification and authorization systems. Equal error rate describes the point at which the false rejection rate (FRR) and false acceptance rate (FAR) are equal. The lower the equal error rate value, the higher the accuracy of the biometric system. The definitions of FAR and FRR are as follows:
  (1) $$FAR=\frac{FP}{FP+TN},$$
  (2) $$FRR=\frac{FN}{FN+TP},$$
  where TP (True Positives) and FP (False Positives) denote the number of true and false owner signature detections, TN (True Negatives) and FN (False Negatives) the number of true and false forgery detection.
- AUC—the area under the Receiver Operating Characteristic Curve (ROC). ROC is a plot that presents TP in the function of FP.

**Figure 5:**
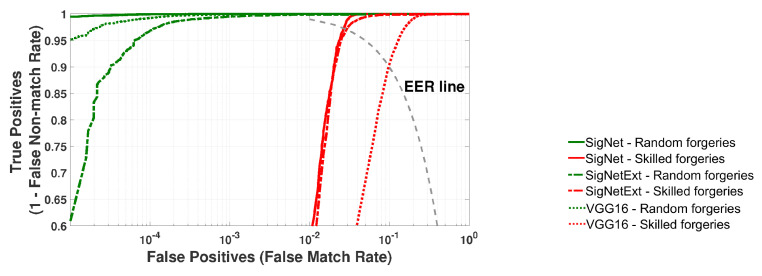
Validation of SigNet, SigNetExt and VGG-16 on the TrainSet dataset. Averaged ROC curves for 20 rounds of cross-validation.

**Figure 6:**
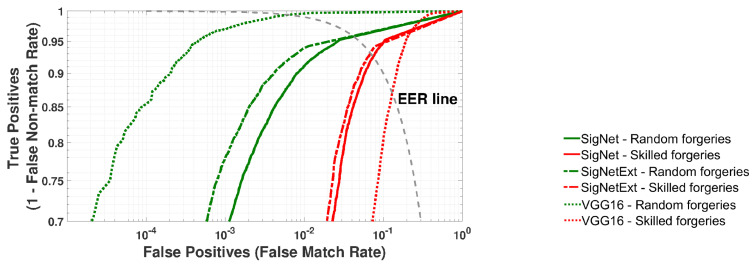
Validation of SigNet, SigNetExt and VGG-16 on the ValSet dataset. Averaged ROC curves for 20 rounds of cross-validation.

The results of the validation of three network models are presented in Figure 5 and Figure 6, and Table 2. All performance measure values average 20 rounds of cross-validation.

Figure 5 depicts the ROC curves calculated based on the recognition results of signatures from the training dataset.

Figure 6 depicts the ROC curves calculated based on the recognition results of signatures from the validation datasets. The goal was to classify all samples from a given ValSet, i.e., signatures that were not used in classifier training.

All experiments confirmed that the accuracy of signature recognition depends on the type of forgeries. Skilled forgeries are more challenging to detect. However, both SigNet architecture classifiers have proven to be quite effective in identifying skilled forgeries.

The SigNet model was the best in identifying random forgeries when the validation procedure was performed on training datasets (TrainSet). However, the situation changed when a validation set (ValSet) was used to check the accuracy of all models. In these experiments, SigNet gave the worst results. It could be caused by data overfitting SigNet. The overfitting problem was reduced by increasing the training dataset with augmentations. SigNetExt seems more generalizable and performs better on the validation set for both scenarios.

## 5. Performance Evaluation of Signature Biometrics

### 5.1. Experiment Setup

Similar to training and validation, all classifiers were tested 20 times for subsets randomly selected from the *MobiBits* database to generalize the results. The scenario of each test was as follows. The user claimed that his signature was class *k*, k∈{1,…,M}, where *M* was the number of users. The acquired sample was preprocessed and entered using the SigNet, SigNetExt, or VGG-16 classifier. Then, the classifier returned the vector of similarities to all *M* classes. The similarity value for class *m* was compared to the acceptance threshold predefined in the system. The user was authorized when the similarity value exceeded the threshold value. The testing procedure was according to the Algorithm 1.


**Algorithm 1** Classifiers testing process.
1:*m* – user identifier, m=1,…,M;2:$S_{i}=\left(sign_{i},k\right)$ – a sample ($sign_{i}$ – signature, *k* – claimed class);3:*P* – vector of probabilities of belonging $sign_{i}$ to *M* classes;4:$T_{a}$ – acceptance threshold value;5:**for** all samples from the testing set (i=1,…,K) **do**6:   Select $S_{i}=\left(sign_{i},k\right)$. Enter $sign_{i}$ at the input of the classifier7:   Check the classifier output *P*;8:   **if** $P\left[k\right]\geq T_{a}$ and $P\left[k\right]=max_{m\in M}P$ **then**9:       positive user verification;10:   **else**11:     forgery detection;12:   **end if**13:
**end for**




### 5.2. Results of Experiments

**Figure 7:**
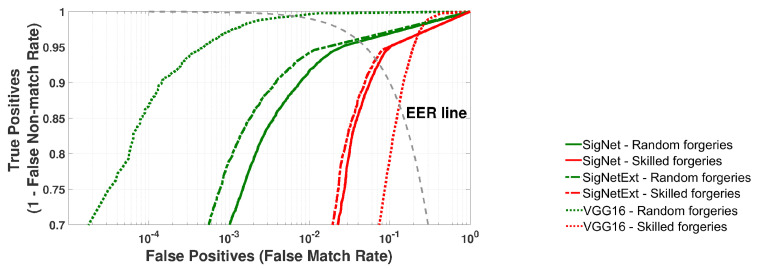
Testing of SigNet, SigNetExt, and VGG-16 on the TestSet dataset. Averaged ROC curves for 20 rounds of cross-validation.

The metrics described in Section 4 were used for the performance evaluation of SigNet, SigNetExt, and VGG-16 classifiers. Figure 7 presents the average ROC curves calculated based on 20 rounds of experiments for the testing datasets (TestSet) randomly selected input datasets from the DataSet. The average EER values and their standard deviations are collected in Table 3. The goal was to classify all samples from a given test set, i.e., signatures that were not used in classifier training and validation.

It can be seen that similar to the results of network model validation presented in Section 4, the equal error rate (EER) for random forgeries is small and similar for all models of classifiers. The differences are relatively small, i.e., average values of EER from 0.63% to 2.70%. The best result, i.e., the smallest ERR, was obtained for the VGG-16 model and was in the [0.34%, 0.92%] range. However, for the SigNet network architecture, the signature recognition accuracy increased after the training dataset’s augmentation (the SigNetExt model). Thus, it can be expected that for a larger size of this dataset, the results will be similar to the VGG-16 classifier. To verify our supposition, we plan to gather more signatures from the potential users to extend our training dataset and train our model on the extended database.

It should be noted that in handwritten online signature biometrics, the immunity to skilled forgeries evaluates the quality of the identity verification system. The immunity is crucial because the modality of a handwritten signature is easily observable and could be quickly replicated by a third party. Thus, the results for skilled forgeries are determinative. That implies that the results for skilled forgeries, i.e., average values of EER from 6.66% to 12.9%, are the main distinctive factor among proposed classifiers. As can be observed, similarly to the results of model validation on TrainSet and ValSet collected in Table 2, the best results were achieved using the SigNetExt classifier. Several issues could explain the poor performance of VGG-16 architecture. Transfer learning and a wide range of parameters compared to the amount of training data might significantly influence the results.

Moreover, initially, VGG-16 was trained to classify diverse images into 1000 different classes. Our problem was less complicated. The VGG-16 network architecture was trained to classify only the gray-scale images of signatures into 53 classes. Utilizing this network in the problem of identity verification with online signatures may require modification of training datasets or more image preprocessing steps.

In conclusion, the experimental results confirm the high efficiency of classifiers implementing CNN architectures. The SigNet, SigNetExt, and VGG-16 models outperformed the widely used online handwritten signature recognition systems employing DTW. The paper [27] reports the results of signatures from the database *MobiBits* verification using DTW. The best EER for skilled forgeries was equal to 18.44%, while the worst result of CNN models was 14.16% (VGG-16|) and 7.31% (SigNetExt). Thus, the SigNet architecture trained on augmented datasets can be recommended for online signature verification on mobile devices.

## 6. Conclusions

With the prevalence of signature forgery as a significant issue in various fields, such as banking, education, and legal documentation, developing an efficient and accessible verification system is crucial. By providing users with a convenient and reliable means of verifying signatures, the mobile application mitigates the risks associated with signature forgery and enhances security in various domains.

This paper presents a study on biometric recognition methods that verify a person’s identity by analyzing online handwritten signatures acquired from mobile devices. We proposed algorithms employing convolutional neural networks for signature verification. Our network architectures were trained, validated, and tested on genuine signatures acquired with mobile devices and collected in the database *MobiBits*. We compared the accuracy of three CNN models and two types of forgeries. This comparison concludes that extending input datasets consisting of coordinates with pressure values may significantly impact the results. Another conclusion is that integrating CNN-based signature verification technology into a mobile application significantly advances document authentication. By providing users with a convenient, reliable, and accessible means of verifying signatures, the mobile application mitigates the risks associated with signature forgery and enhances security in various domains.

This paper creates the base for many research studies and future experiments. In our future work, we plan to train and evaluate our network models on more diverse datasets and input data in different forms. Moreover, we plan to design and test other CNN architectures and perform a comprehensive comparison with other existing solutions for online handwritten signature verification on mobile phones.

The second research area under consideration is to create a dataset with generated forgeries and train neural network models on training datasets containing genuine and forged signatures.

## Figures and Tables

**Table 1:** The summary of SigNet and VGG-16 network architectures.

	SigNet	VGG-16
size of input data	77 × 190	224 × 224
layers	4 convolutional layers (with ReLU and pooling) 1 fully connected softmax	13 convolutional layers with ReLU and ax pooling 3 fully connected softmax
sizes of filters	9×9, 7×7, 5×5, 6×16	3×3
number of filters	32, 32, 64, 64	64, 128, 256, 512
optimization	SGD, m=0.9	SGD, m=0.9
learning rate	0.05–0.0005	0.0001
training	trained on online signatures	trained on natural images and fine-tuned using transfer learning on online signatures
regularization	data augmentation	data augmentation 50% dropout

**Table 2:** Mean Equal Error Rates (EER) for signature recognition and random and skilled forgeries. Validation of classifiers on training and validation datasets.

Input Dataset	Classifier	Random Forgeries	Skilled Forgeries
	SigNet	**0.01 ± 0.01%**	**2.66 ± 0.41%**
TrainSet	VGG-16	0.10 ± 0.10%	9.77 ± 1.37%
	SigNetExt	0.25 ± 0.12%	2.88 ± 0.37%
	SigNet	2.81 ± 0.36%	7.47 ± 0.67%
ValSet	VGG-16	**0.63 ± 0.38%**	12.47 ± 1.39%
	SigNetExt	1.14 ± 0.19%	**6.88 ± 0.76%**

**Table 3:** Mean Equal Error Rates (EER) for signature recognition and random and skilled forgeries. Evaluation of classifiers on the testing dataset (TestSet).

CNN Architecture	Random Forgeries	Skilled Forgeries
SigNet	2.70 ± 0.29%	7.16 ± 0.75%
VGG-16	**0.63 ± 0.29%**	12.90 ± 1.26%
SigNetExt	1.14 ± 0.17%	**6.66 ± 0.65%**

## Data Availability

Data supporting reported results are available on request from the corresponding author.

## Abbreviations

The following abbreviations are used in this manuscript:

EER	Equal Error Rate
DTW	Dynamic Time Warping
FAR	False Acceptance Rate
FRR	False Rejection Rate
PCA	Principal Component Analysis
CNN	Convolutional Neural Network
RNN	Recurrent Neural Network
TA-RNN	Time-Aligned Recurrent Neural Network
